# Assessment of noninvasive liver stiffness in inactive HBsAg carriers by transient elastography: Fibroscan in inactive HBsAg carriers

**Published:** 2011-03-01

**Authors:** Ioan Sporea, Diana Nicolita, Roxana sirli, Alexandra Deleanu, Adriana Tudora, Simona Bota

**Affiliations:** 1 Department of Gastroenterology and Hepatology, Victor Babes University of Medicine and Pharmacy, Timisoara, Romania

**Keywords:** Transient, Elastography, Liver, HBsAg

## Abstract

**Background:**

Chronic viral hepatitis can be evaluated using invasive or noninvasive methods.

**Objectives:**

The aim of this study was to evaluate liver stiffness in inactive HBsAg carriers compared with normal subjects and determine if it is influenced by viral load in these patients.

**Patients and Methods:**

We prospectively evaluated 140 inactive HBsAg carriers and 152 normal subjects (without any signs or history of liver disease). In all subjects, liver stiffness was measured by 3 experienced physicians using a FibroScan® device (EchoSens, France) per standard procedures. We excluded patients for whom the SR of liver stiffness measurements was < 60% and those who had measurements with an IQR >30%.

**Results:**

The mean liver stiffness in inactive HBsAg carriers was 5.6±2.1kPa, significantly higher than in normal subjects (4.8 ± 1.2 kPa, p = 0.0002). In 16.4% (23) of inactive carriers, liver stiffness exceeded 7 kPa (the cutoff for significant fibrosis F ≥ 2). In patients with undetectable viral loads, the mean liver stiffness was 4.9 ± 1.2 kPa, significantly lower than in those with detectable DNA (< 2000 IU/ml) (6.7 ± 2.7 kPa, p < 0.001).

**Conclusions:**

Inactive HBsAg carriers have higher liver stiffness values compared with healthy individuals. Liver stiffness in inactive HBsAg carriers with detectable viral loads is higher than in those who are aviremic, suggesting that low viral loads promote fibrosis.

## Background

Worldwide, chronic hepatitis B virus (HBV) infection is a significant public health problem; approximately 350,000,000 people are estimated to be infected with this virus. Some patients have chronic hepatitis or cirrhosis, whereas others are inactive carriers. Yet, it is possible that viral replication restarts in inactive HBsAg carriers, resulting in the development of chronic hepatitis. In the past, nearly all patients with chronic HBV hepatitis were evaluated by liver biopsy (LB) to determine the stage and grade of the disease. Today, few patients undergo LB (those with less than a 2-fold increase in aminotransferase levels) to determine whether the liver lesions (especially necroinflammation) are severe enough to justify treatment.

Typically, inactive HBsAg carriers are not examined through invasive procedures, such as LB. Instead, liver lesion severity can be assessed noninvasively in inactive HBsAg carriers and chronic HBV hepatitis patients by transient elastography (TE) or biological assay (e.g., FibroTest-ActiTest). In recent years, new noninvasive methods of evaluation have become available, such as liver stiffness (LS) measurement by means of TE (FibroScan®) or biological tests (FibroTest-ActiTest®). These methods are tolerated easily by the patient and have good reproducibility [1]. Further, they are not influenced by the subject's age [2] and, according to some studies, yield similar results as percutaneous LB [3][4]. After 2000, noninvasive tests were used more frequently, particularly with the recognition of TE for the evaluation of patients with chronic HCV hepatitis [5]. Recently, algorithms have been developed to assess chronic HBV hepatitis and nonalcoholic steatohepatitis.

There is a robust correlation between LS measurements (FibroScan) and fibrosis, as diagnosed by LB, in HBV cirrhosis [4][6][7][8]. A meta-analysis of 7 studies demonstrated that TE has good predictive value for significant fibrosis [9], with 70% sensitivity (95% CI: 67% to 73%), 84% specificity (95% CI: 80% to 88%), a positive likelihood ratio of 4.2 (95% CI: 2.4-7.2), and a negative likelihood ratio of 0.31 (95% CI: 0.23-0.43). Moreover, the advantages of TE in evaluating inactive HBsAg carriers are that it detects the possible reactivation of disease and facilitates the diagnosis of other causes that can lead to liver injury [10][11] for instance, concomitant alcohol abuse. Nevertheless, TE has disadvantages-the most significant of which is that liver stiffness (LS) can be influenced by factors other than liver fibrosis, such as ALT flares [12].

## Objectives

The aims of this report were to evaluate LS in inactive HBsAg carriers compared with normal subjects and determine whether LS is influenced by viral load in these patients.

## Patients and Methods

### Patients

Our study group comprised 140 prospectivelly included, inactive HBsAg carriers. The diagnosis of inactive HBsAg carrier was based on the following criteria: persistent normal ALT and AST serum levels (at least 3 measurements during a 6-month period), positive HBsAg, negative HBeAg, and HBV DNA viral load < 2000 IU/ml (< 10,000 copies/ml). The viral load was measured by real-time PCR (m2000sp/m2000rt Abbott), with a detection limit of 51 copies HBV DNA/ml (1.71 log copies HBV DNA/ml). None of the patients was coinfected with HCV, HDV, or HIV. All patients were evaluated in the Department of Gastroenterology and Hepatology, University of Medicine, Timişoara. The control group included 152 normal subjects-healthy volunteers or patients from other departments in our hospital (e.g., nephrology, surgery, cardiology). The healthy volunteers (109 individuals) were medical students, nurses, and medical doctors (fellows and specialists) from our hospital. None had a history of liver disease (acute or chronic). At the TE evaluation, we did not perform additional tests in this subgroup, such as biological tests, viral markers, and abdominal ultrasonography.

The hospitalized patients (43 individuals) had no history of liver disease (acute or chronic), had normal aminotransferase levels, and did not exhibit stetatosis by ultrasonography. Also, none had a severe disease, such as congestive heart failure, renal failure, and diabetes. Their chief diagnoses included acute pyelonephritis, appendicitis, chronic glomerulonephritis, arterial hypertension, and chronic angina pectoris. All subjects agreed to participate in this study, which was approved by the local ethical committee.

### Transient elastography

LS measurements (LSMs) were taken by 3 experienced physicians (each performing more than 800 TE evaluations) by TE in all patients using a FibroScan® device (EchoSens - Paris, France). The intraobserver reproducibility of TE in our center has been high [13], yielding intraclass correlation coefficients (ICCs) of 0.985, 0.949, and 0.874 for the 3 examiners, respectively. Also, the interobserver reproducibility was excellent for the examiners, with an overall ICC of 0.982. The measurements were made with the patients lying in the dorsal decubitus position with the right arm in maximal abduction. The right lobe of the liver was aimed at through the intercostal spaces. The tip of the probe transducer was covered with coupling gel and placed on the skin, between the ribs at the level of the right lobe of the liver. The operator, assisted by time-motion ultrasonography and A-mode images that were provided by the system, located a portion of the liver that was free of large vascular structures and at least 6 cm thick. Once the measurement area was identified, the operator pressed the probe button to begin an acquisition. Ten successful acquisitions were performed for each patient, and the median value was calculated by the device, expressed in kiloPascals (kPa) [14]. The success rate (SR) was calculated as the ratio of the number of successful acquisitions to the total number of acquisitions. Only patients in which 10 LSMs were obtained with an SR of at least 60% and with an IQR < 30% (IQR = interquartile range-the difference between the 75th and 25th percentiles, essentially the range of the middle 50% of the data) were considered to be reliable [15]. Failure was defined as a case in which 10 valid measurements could not be obtained. Failures and those with unreliable LSMs were not included in the analysis. LSM failures were caused primarily by abdominal obesity and narrow intercostal spaces [15][16].

### Statistical analysis

The data were collected in a Microsoft Excel file. The statistical analysis was performed using Microsoft Excel and GraphPad Prism. The mean and standard deviation were calculated for the quantitative variables. Unpaired t-test was used to compare means. Associations between assay results and viral load in inactive HBsAg carriers were analyzed using the Spearman rank correlation coefficient (r).

## Results

**Figure 1:**
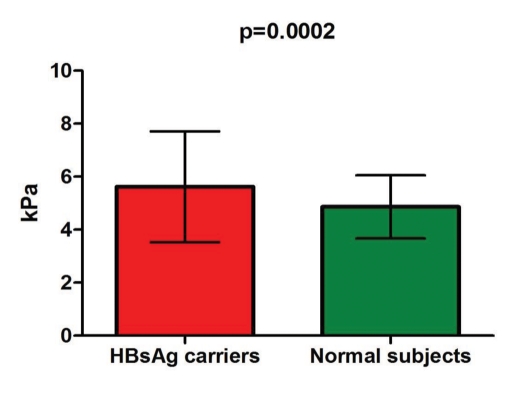
Mean values of LS in normal subjects and HBsAg carriers

**Figure 2:**
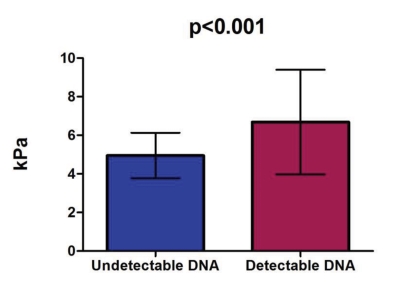
Mean LS values in patients with undetectable viral loads versus patients with detectable viral loads below 2000 IU/ml

**Figure 3:**
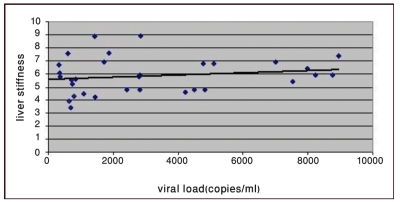
Relationship between LS and viral load in inactive HBsAg carriers (Spearman r = 0.163, p = 0.365)

The study group comprised 140 inactive HBsAg carriers, and the control group included 152 normal subjects; their demographic data are shown in Table 1. The mean LS value in the 140 inactive HBsAg carriers was 5.6 ± 2.1 kPa compared with 4.8 ± 1.2 kPa in the normal subjects (p = 0.0002), as seen in Figure 1. In 16.6% of cases (20 inactive HBsAg carriers), the LS exceeded 7 kPa (the cutoff for significant fibrosis), requiring further investigation to determine the cause. One HBsAg carrier had an LS of 20.1 kPa (indicating liver cirrhosis). Excluding this patient, the mean LS was 5.5 ± 1.7 kPa, significantly higher than in normal subjects (4.8 ± 1.2 kPa) (p = 0.00017). The mean LS in patients with undetectable viral loads (86 patients) was 4.9 ± 1.2 kPa, lower than in patients with detectable DNA levels (under 2000 IU/ml- 54 patients) (6.7 ± 2.7 kPa, p < 0.001) (Figure 2). In determining whether viral load in HBsAg carriers correlated with LS, we observed that LS increased in patients with higher viral loads (under 2000 IU/ml), but this result was not significant (Spearman r = 0.163, p = 0.365) (Figure 3).

**Table 1:** Demographics of HBsAg carriers and normal subjects

**Parameter**	**Inactive HBsAg carriers**(Mean ± SD)	**Normal subjects**(Mean ± SD)	**p-value**
**Age (years)**	38.7 ± 12.9	43.5 ± 15.9	0.0055
**Male**	69/140 (49.3%)	65/152 (42.8%)	0.2909
**Body mass index(kg/m2)**	25.15 ± 14.27	24.34 ± 4.7	0.8586

## Discussion

In this study, we determined whether TE was useful for noninvasive evaluation of liver fibrosis in inactive HBsAg carriers. Hilleret et al. [17] examined 117 consecutive inactive HBsAg carriers during a 4-year period (2003-2007). They defined inactive HBsAg carrier status as follows: HBsAg+; HBeAg-; anti-HBe +; normal ALT at least on 3 occasions during a minimal 6-month follow-up; HBV DNA lower than 4000 IU/ml; anti-HCV, and anti-delta-negative. These inactive carriers were compared with 174 patients with histologically proven HBeAg- chronic hepatitis, evaluated in the same period. In each patient, LS was measured by FibroScan®. In inactive carriers, the mean LS was 4.9 kPa versus 8.6 kPa in patients with histologically documented HBeAg- chronic hepatitis (p < 0.001). Further, the mean LS in inactive carriers (excluding inactive cirrhosis) was compared with that of patients with mild HBeAg- chronic hepatitis (F0/F1 Metavir, N = 87). LS was significantly lower in inactive HBsAg carriers (4.9 vs. 8.1 kPa, respectively; p < 0.03). Also, in 95.6% of inactive carriers, LS was lower than 7 kPa, confirming the absence of significant fibrosis.

In our study, the mean LS in inactive carriers was 5.7 kPa, compared with 4.9 kPa in the Hilleret study. Several studies have been published regarding the upper limit of normal LS values. In Chu et al. values under a cutoff of 6 kPa were highly predictive of insignificant fibrosis in patients with normal ALT levels [18]. The cutoff was higher, 7.5 kPa, when ALT was elevated (up to 5 times the upper limit of normal [ULN]). In Roulot et al. [16], the proposed LS values for normal livers were 3.3-7.8 kPa in women and 3.8-8.0 kPa in men; in Corpechot et al. [19], the LS values ranged from 2.5-6.9 kPa. In a study by our group, the mean LS in 144 normal subjects was 4.8 ± 1.3 kPa [2]. Yet, Chan et al. [6], Oliveri et al. [10], and Coco et al. [12] demonstrated that LS values that are obtained by TE are influenced by transaminase levels and that this influence is absent in inactive carriers due to the persistent normal values of ALT in these subjects. Marcellin et al. [4] observed that TE adequately assesses LS in patients who are infected with HBV. In their study, which included 173 patients for whom both liver biopsy and TE were performed, LS correlated significantly with Metavir (r = 0.65) and Ishak fibrosis stage (r = 0.65) (p < 0.001). The areas under the receiver-operating characteristic curves (AUROCs) were 0.81 (95% CI: 0.73-0.86) for F ≥ 2, 0.93 (95% CI: 0.88-0.96) for F ≥ 3, and 0.93 (95% CI: 0.82-0.98) for F = 4. The optimal LS cutoff values were 7.2 and 11.0 kPa for F ≥ 2 and F = 4, respectively. The authors concluded that LS reliably detects significant fibrosis and cirrhosis in HBV patients and that the cutoffs differ slightly from those in HCV patients. In inactive HBsAg carriers, one must determine whether their status is permanent or whether they require long-term follow-up.

Viral replication can restart in certain inactive carriers, causing chronic hepatitis (or cirrhosis) to develop. In Chu et al. [18], 1965 inactive HBsAg carriers were evaluated (mean age 35.6 years; 1076 males). During a mean follow-up of 11.5 years, HBV hepatitis reactivated in 314 carriers (13%) (defined as ALT flares more than twice the upper limit of normal and positive HBV DNA by hybridization assay). The risk of HBV reactivation correlated significantly with older age on entrance into the study (p < 0.0001) and male gender (p < 0.0001). Fifty-seven patients developed cirrhosis, for which the cumulative incidence was 15% after 25 years. The risk of cirrhosis correlated significantly with advanced age at entry (p = 0.004) and HBV reactivation (p < 0.0001). Of the 1651 carriers who did not experience HBV reactivation, 10 developed cirrhosis, and the only significant factor was advanced age at entry (p=0.03). Of the 314 patients with HBV reactivation, cirrhosis developed in 47, and the cumulative incidence was 8%, 16%, 27%, and 46% at 5, 10, 15, and 20 years after reactivation. Male gender (p = 0.037) and advanced age at reactivation (p = 0.006) were independent risk factors. Chu et al. concluded that the so-called inactive carrier state cannot be considered generally as an innocuous, persistent condition with good prognosis, suggesting that regular follow-up is necessary. Based on these findings, we believe that regular follow-up of inactive HBsAg carriers by sequential viral load measurements (every 3-6 months) and annual LS measurements constitute a reasonable workup for this category of patients. In our study, one patient had a high LS values (20.1 kPa) and no biological, endoscopic, or ultrasonographic signs of cirrhosis. A liver biopsy was required to determine whether the patient had inactive cirrhosis, but he refused to undergo the procedure. We found that inactive HBsAg carriers had significantly higher LS values compared with normal subjects (5.6 ± 2.1 kPa vs. 4.8 ± 1.2 kPa, respectively; p = 0.0002). Further, the mean LS in patients with undetectable DNA was 4.9 ± 1.2 kPa, lower than in patients with detectable DNA (but < 2000 IU/ml) (6.7 ± 2.7 kPa, p < 0.001), suggesting that low viral loads promote fibrosis. Although LS values increased in patients with higher viral loads, the relationship was not significant (Spearman r = 0.163, p = 0.365). Inactive HBsAg carriers had higher LS values than normal subjects. The mean LS was lower in patients with undetectable viral loads than in patients with detectable viral loads, suggesting that low viral loads accelerate fibrosis. Inactive HBsAg carriers with alarmingly high LS values (16.6% of patients had LS >7 kPa) require further examination, including liver biopsy. TE (FibroScan) is a good, noninvasive method that can be used to follow up these patients to assess the progression of fibrosis.
